# The Role of Natural Products in Diabetic Retinopathy

**DOI:** 10.3390/biomedicines12061138

**Published:** 2024-05-21

**Authors:** Yuxuan Zhao, Yi Chen, Naihong Yan

**Affiliations:** 1 Research Laboratory of Ophthalmology, West China Hospital, Sichuan University, Chengdu 610041, China; zhaoyx413@163.com (Y.Z.); chenyicycycy0723@gmail.com (Y.C.); 2 Department of Optometry and Visual Science, West China Hospital, Sichuan University, Chengdu 610041, China

**Keywords:** diabetic retinopathy, natural products, mechanism, toxicity

## Abstract

Diabetic retinopathy (DR) is one of the most severe complications of diabetes mellitus and potentially leads to significant visual impairment and blindness. The complex mechanisms involved in the pathological changes in DR make it challenging to achieve satisfactory outcomes with existing treatments. Diets conducive to glycemic control have been shown to improve outcomes in diabetic patients, thus positioning dietary interventions as promising avenues for DR treatment. Investigations have demonstrated that natural products (NPs) may effectively manage DR. Many types of natural compounds, including saponins, phenols, terpenoids, flavonoids, saccharides, alkaloids, and vitamins, have been shown to exert anti-inflammatory, antioxidant, anti-neovascular, and antiapoptotic effects in vivo and in vitro. Nevertheless, the clinical application of NPs still faces challenges, such as suboptimal specificity, poor bioavailability, and a risk of toxicity. Prospective clinical studies are imperative to validate the therapeutic potential of NPs in delaying or preventing DR.

## 1. Introduction

Diabetes mellitus (DM) is a metabolic disorder characterized by chronic hyperglycemia due to a variety of etiological factors. A survey by the International Diabetes Federation indicated that in 2021, approximately 537 million individuals aged 20–79 years were diagnosed with DM. Diabetic retinopathy (DR) is a severe complication of DM, and persistent hyperglycemia and hypertension can lead to retinal dysfunction, thus ultimately resulting in significant visual impairment and even blindness [1]. Factors such as hyperglycemia, hypertension, dyslipidemia, and duration of DM are associated with increased risks of DR [2,3]. By 2045, DR is expected to affect an estimated 1.6 million people worldwide [4].

**Figure 1:**
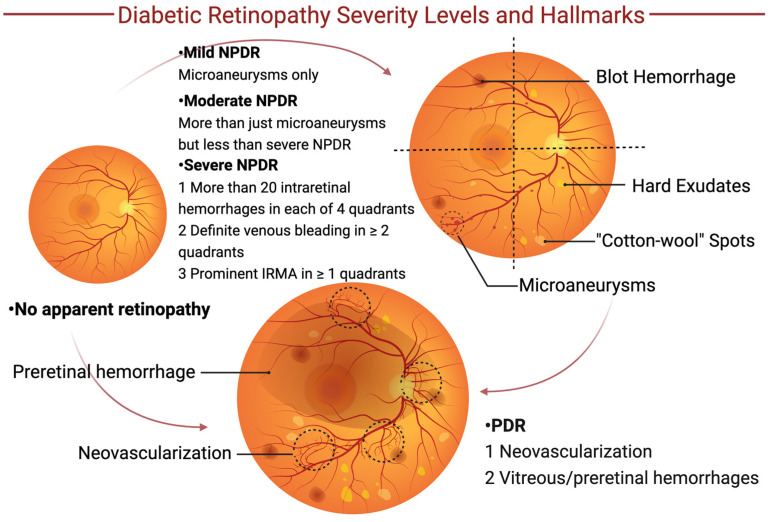
Diabetic retinopathy severity levels and hallmarks. Initially, diabetic patients may exhibit no apparent signs of retinopathy. However, as diabetic retinopathy (DR) progresses, it may evolve into non-proliferative diabetic retinopathy (NPDR) and eventually advance to proliferative diabetic retinopathy (PDR), or it may transition directly to PDR. According to international classifications, NPDR is categorized into mild, moderate, and severe stages. Clinical manifestations of NPDR include blot hemorrhages, hard exudates, “cotton-wool” spots, and microaneurysms. The more advanced stage, PDR, is characterized by the emergence of neovascularization and/or vitreous or preretinal hemorrhages. Abbreviations: IRMA, intraretinal microvascular abnormalities.

DR can be divided into two stages: non-proliferative diabetic retinopathy (NPDR) and advanced proliferative diabetic retinopathy (PDR) [5]. As the early stage of DR, NPDR may present without subjective symptoms, yet the fundus exhibits pathological alterations, such as microaneurysms, hemorrhages, cotton-wool spots, and hard exudates. PDR, which is a progressive stage of NPDR, is mainly associated with persistent hyperglycemia and inadequate glycemic control [6]. This advanced stage is characterized by retinal microvascular damage and pericyte loss, thus manifesting as vitreous or preretinal hemorrhages and neovascularization (Figure 1).

The pathogenesis of DR is very complex because it involves multiple cross-linking mechanisms, which lead to retinal dysfunction [7]. Current treatments for DR include medications to control blood glucose levels, retinal laser photocoagulation, anti-vascular endothelial growth factor (VEGF) injection, and para plana vitrectomy (PPV). Although VEGF inhibitors and other antiangiogenic agents have been widely used in the clinical settings, many patients still do not achieve satisfactory visual recovery [8]. Consequently, the identification of novel therapeutic strategies to ameliorate DR is urgently needed.

Currently, the rapidly developing economic model has changed individuals’ lifestyles and dietary patterns. An increased consumption of sugar and fats, as well as diminished physical activity, may exacerbate DM. Diet and lifestyle modifications constitute the foundational strategy for DR management. Natural products (NPs) derived from various fauna and flora have emerged as being dietary drug supplements and have increasingly become a focus of research interest. One study demonstrated that increasing daily fruit consumption may mitigate DR risks, with a notable 50% reduction attributed to the abundant vitamins and nutrients in fruits [9]. The benefits of a wide range of vegetables and fruits reinforce the shift toward dietary-based therapeutic modalities.

This review summarizes and updates the current evidence on the possible role of NPs in DR, ranging from experimental findings to the possible challenges that may be encountered in clinical application; moreover, we expect to introduce a novel therapeutic method for DR patients.

## 2. Method

The search strategy was based on the Preferred Reporting Items for Systematic Reviews and Meta-Analyses (PRISMA) guidelines [10]. Studies related to diabetic retinopathy and natural products were collected through searches on the Web of Science and PubMed, from January 1974 to January 2024. The keywords “Diabetic Retinopathy”, “Biological Products”, and “Natural Products” were utilized, combined as Mesh terms and text words. Boolean operators were used to construct the search strategy. Relevant reference lists were also searched, ensuring that all relevant literature was involved.

Inclusion criteria:

1. Natural extracts from plants, animals, or medical herbs were considered.
2. The effectiveness of natural products in treating diabetic retinopathy was validated through in vitro, in vivo, or human clinical trials.
3. The results included effects, therapeutic potentials, signaling mechanisms, etc.

Exclusion criteria:

1. Studies not relevant to natural products were excluded.
2. Natural products lacking clear chemical structures were omitted.
3. Natural products with multiple components that did not specify the active components were excluded.

## 3. Diabetic Retinopathy

### 3.1. Pathophysiology of Diabetic Retinopathy

The pathophysiologic changes in DR are associated with chronic hyperglycemia. Persistent elevation of blood glucose is implicated in physiological and biochemical retinal alterations, thus precipitating microvascular injury and retinal dysfunction. Moreover, chronic hyperglycemia is known to induce microaneurysms, hemorrhages, and thickening of the retinal basement membrane, which increase the permeability of the blood–retina barrier (BRB) and cause leakage from retinal vessels. Concurrently, compromised vascular permeability contributes to capillary occlusion and subsequent retinal hypoxia, thus potentially leading to increased VEGF levels [11] and fostering PDR [3]. PDR is pathologically characterized by the emergence of retinal neovascularization and fibrovascular membranes, with potential progression to vitreous hemorrhage and retinal detachment, thus culminating in visual impairment and blindness. Diabetic macular edema (DME) represents another prevalent cause of visual loss in DR and is characterized by disruption of the BRB and subsequent accumulation of fluid in the macula, thus resulting in increased macular thickness and edema [12]. DME may occur at any stage of DR and may cause severe image distortion and vision loss. Additionally, indicators such as inflammation, micro-vasculopathy, oxidative stress, and neurodegeneration have been implicated in DR-related retinal damage.

#### 3.1.1. Inflammation

Inflammation plays a pivotal role in all stages of DR. Inflammatory factors can be consistently detected at low doses in diabetic patients and animal models [13,14]. Adhesion-molecule-mediated leukocyte-endothelial cell adhesion is associated with leukostasis in DR, and it has been proven that leukostasis is correlated with endothelial cell loss and BRB damage in diabetic models [15]. In addition, increased expression of leukocyte adhesion molecules and endothelial cell adhesion molecules has been detected in diabetic patients and animal models [16,17].

Furthermore, chemokines, such as monocyte chemotactic protein-1 (MCP-1), macrophage inflammatory protein-1α (MIP-1α), and MIP-1β, are involved in DR progression [18]. These chemokines can attract and activate leukocytes, thus exacerbating leukostasis. The expression of inflammatory mediators, including tumor necrosis factor-alpha (TNF-α), interleukin 6 (IL-6), IL-10, IL-23, and IL-1β, is also upregulated in DR patients [19].

Microglial activation is involved in the inflammatory response in DR. Under high-glucose conditions, microglia are activated, followed by increased levels of TNF-α, IL-6, MCP-1, and VEGF [20]. Müller cells and astrocytes also contribute to this response, thus releasing large amounts of pro-inflammatory factors that further exacerbate inflammation in the retina [21].

#### 3.1.2. Retinal Micro-Vasculopathy

DR has long been recognized as a microvascular disease [22]. Chronic hyperglycemia is known to induce vasodilatation and hemodynamic changes, which potentially occur as a retinal adaptation to altered metabolic demands [23]. In DR, systemic and ocular hypertension, in addition to hyperglycemia, contribute to the disruption of tight junctions between pericytes and endothelial cells. This disruption leads to pericyte apoptosis, which precipitates the uncontrolled proliferation of vascular endothelial cells and new vessel formation [24]. Due to the fact that pericytes support capillary integrity, their loss is implicated in the pathogenesis of capillary dilation and subsequent microaneurysm development [25]. Moreover, the newly formed vasculature, which tends to be delicate and excessively permeable, can lead to leakage, thus resulting in hemorrhage and swelling in the retina [26].

Retinal ischemia or hypoxia stimulates hypoxia-inducible factor-1 (HIF-1), thus stimulating VEGF upregulation [27]. VEGF has been implicated as a principal factor in the pathogenesis of PDR and DME. VEGF can increase vascular permeability by phosphorylating tight-junction proteins [28]. Furthermore, VEGF facilitates endothelial cell proliferation via mitogen-activated protein (MAP) activation [29]. Elevated VEGF levels have been detected in DR patients and animal models [30,31,32].

#### 3.1.3. Oxidative Stress

Oxidative stress is a key factor contributing to the diminished capacity of intracellular antioxidant defense systems and fostering pro-inflammatory factor production [33]. The excessive accumulation of reactive oxygen species (ROS) precipitates oxidative stress. Under physiological conditions, organisms resist ROS through the antioxidant defense system, thus maintaining a balance between ROS generation and elimination [34]. However, chronic hyperglycemia leads to decreased antioxidant defenses and increased oxidative stress [35]. Hyperglycemia activates metabolic pathways, stimulates mitochondrial oxidative phosphorylation, and activates nicotinamide adenine dinucleotide phosphate oxidase, all of which contribute to increased ROS levels [36]. Elevated ROS levels may alter cellular homeostasis, thus leading to cellular dysfunction. In addition, the retina is particularly susceptible to oxidative stress, considering its high unsaturated fatty acid content and substantial oxygen consumption for glucose metabolism. In diabetic mouse models, a significant increase in ROS production and concomitant suppression of antioxidant enzyme activity were observed [37].

#### 3.1.4. Retinal Neurodegeneration

Retinal neurodegeneration may occur early in DR, and upregulated expression of pro-apoptotic factors (such as cleaved caspase-3, Bax, and Fas) has been detected in DR patients and animal models [38,39,40]. Studies have indicated that sustained exposure to high glucose levels is correlated with increased mitochondrial fragmentation and apoptosis in vitro [41]. Observations in diabetic mouse models demonstrated ganglion cell loss and reduced retinal thickness, which precede the onset of retinal micro-vasculopathy [42].

### 3.2. Current Therapy for Diabetic Retinopathy

Current treatment modalities for DR include medications, anti-VEGF injections, laser photocoagulation, and PPV [8]. Nevertheless, the available methods for treating DR remain unsatisfactory. The optimal management of blood glucose and diabetic progression remains of utmost importance [43]. In the stages of normal or mild to moderate NPDR without macular edema, glycemic control is typically achieved through pharmacological methods, which include the use of insulin-promoting agents, such as sulfonylureas, glinides, dipeptidyl peptidase IV inhibitors, and hypoglycemic agents acting through other mechanisms, such as bisphosphonoids, thiazolidinediones, α-glycosidase inhibitors, and sodium-glucose cotransporter protein 2 inhibitors. Concurrently, the risk of diabetic retinopathy onset and progression can be reduced through the use of antihypertensive drugs (angiotensin-converting enzyme inhibitors, angiotensin II receptor antagonists, and calcium channel blockers) and hypolipidemic drugs (stains, fibrates, and nicotinic acid).

Clinically, anti-VEGF agents, such as faricimab-svoa, ranibizumab, aflibercept, and bevacizumab, are also prevalently used to inhibit retinal neovascularization and mitigate macular edema. Nonetheless, intravitreal anti-VEGF injections have several disadvantages, such as high costs and potential side effects [44]. The short half-life of these drugs necessitates frequent, monthly, or bimonthly administration to sustain therapeutic benefits, and long-term intravitreal treatments increase the risk of complications, such as endophthalmitis, uveitis, and subconjunctival hemorrhage [45]. These issues, including long-term treatment necessity, high costs, and associated risks, may render sustained therapy infeasible for a majority of patients.

Laser photocoagulation serves as an efficacious adjunct therapy for DR patients. The Early Treatment Diabetes Retinopathy Study (ETDRS), which lasted for three years, established that focal/grid macular laser photocoagulation was beneficial for individuals with DME, thus significantly reducing the likelihood of vision loss [46]. Pan-retinal photocoagulation (PRP) has been shown to suppress VEGF production by ameliorating retinal microcirculatory hemodynamics and augmenting the oxygenation of the retina. Despite its efficacy, PRP may induce adverse effects, such as slight central visual loss, vitreous hemorrhage, and subsequent neovascularization. The integration of focal laser and anti-VEGF injections in DME patients can lessen the need for frequent anti-VEGF injections, thereby mitigating complication risks, and has become a popular treatment modality for DR patients [47].

**Figure 2:**
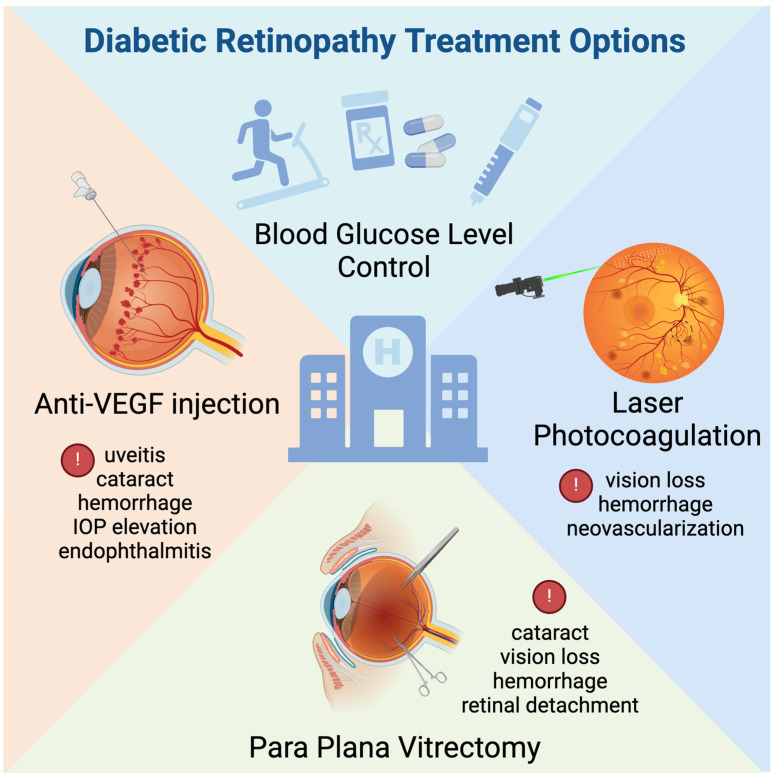
Diabetic retinopathy treatment options. Controlling the blood glucose level is critical, which involves healthy lifestyle, medical management, and insulin injections. Additionally, clinical practices commonly employ anti-vascular endothelial growth factor (VEGF) injections, laser photocoagulation, and pars plana vitrectomy. While these interventions are prevalent, they can present certain adverse effects and complications.

In cases of PDR complicated by vitreous hemorrhage or tractional retinal detachment, PPV is employed as the principal intervention. PPV facilitates the removal of intraocular blood and diminishes the vitreous concentrations of VEGF and inflammatory mediators, thus consequently ameliorating disruptions in the retinal microenvironment. Concurrently, excision of the fibrovascular membrane (FVM) during surgery alleviates traction, thus re-establishing the normal structure of the retina. However, PPV possesses a greater risk of hemorrhage during the operation and presents challenges for achieving successful surgical outcomes. Current treatment options for DR and their side effects can be seen in Figure 2.

## 4. Experimental Models for Diabetes Research

### 4.1. Cell Models

Research utilizing cell models has proven to play an essential role in elucidating the responses of cells in different retinal layers to diabetes-related stimuli, such as high glucose, lipids, shear stress, metabolite imbalance, cytokines, and growth factors [43]. These models facilitate the investigation of the crucial molecular mechanisms that are involved in the interaction between pharmacological agents and DR. In studies of NPs related to DR, endothelial cells and retinal pigment epithelium (RPE) cells are the predominant cell models that are employed, with fewer studies using pericytes, Müller cells, and ganglion cells.

Pathological neovascularization is the leading cause of DR progression, and researchers usually utilize endothelial cells in vitro to stimulate human retinal angiogenesis [48]. NP investigations have employed human retinal endothelial cells (HRECs), rhesus monkey retinal and choroid endothelial cells (RF/6A), human umbilical vein endothelial cells (HUVECs), and human microvascular endothelial cells (HMVECs), the latter two of which serve as non-specific models for angiogenic replication. In vitro, cells are treated with high glucose concentrations to stimulate a diabetic condition. Endothelial cells are commonly utilized to investigate cell proliferation, migration, tube formation, and signaling pathways related to inflammation and apoptosis. Expressions of HIF-1α and VEGF are often assessed in these studies [49,50,51]. However, differences in the secretory functions of HREC and HUVEC and their modulation by glucose suggest regional specificity of cellular functions. Considering that DR is a microvascular disease and is specifically restricted in the retina, the use of HUVECs, a macrovascular cell type, in in vitro studies may be inadequate for inferring pathophysiologic changes in DR [52].

RPE cells, which are located between the retina and the choroid, are crucial for preserving the integrity of photoreceptors in terms of both structure and function [53]. Nevertheless, studies have indicated that DR can lead to RPE cells’ dysfunction and impaired clearance of fluid leakage from choroidal capillaries, thus culminating in retinal edema [54]. Extensive research has demonstrated that high glucose exposure contributes to retinal barrier dysfunction, early apoptosis, and aberrant cytokine expression. NPs have been identified as being protective agents for RPE cells, delivering anti-inflammatory and antioxidant properties [55,56].

Other in vitro studies have utilized specific retinal cells, including glial cells, macrophages, and retinal neuronal cells. The human retina is comprised of three types of glial cells: microglia, astrocytes, and Müller cells. These glial cells are crucial supporters of neuronal tissue, closely associating and interacting with retinal vessels and other structures to maintain the homeostasis of the retinal microenvironment. In vitro studies involving glial cells typically investigate the relationship between DR vasculopathy and neuropathy [57]. The migration of macrophages is closely linked to inflammation and angiogenesis in DR; thus, studies employing macrophages are pivotal in elucidating the underlying mechanisms [58]. Evidence indicates that profound morphological changes and electrophysiologic defects occur in retinal neurons early over the course of DR [59]. Research employing retinal neuronal cells, including photoreceptors and retinal ganglion cells, plays a vital role in understanding the neurodegeneration associated with DR [60].

It must be acknowledged that investigations on the role of NPs in DR through cell models have inherent limitations. The retina is an intricate tissue composed of multiple interrelated cell types, and the isolation of specific cells fails to represent the entirety of retinal microenvironmental alterations [61]. Besides, due to long-term development of diabetic complications, possibly due to the accumulation of epigenetic changes [62], in vitro models with a relatively rapid experimental duration are inferior to animal models. Therefore, animal models are posited to be a more appropriate alternative.

### 4.2. Animal Models

Diabetic animal models have been instrumental in understanding the molecular pathology underlying DR and assessing NPs as potential alternative treatments. Commonly used models in DR research include mice, rats, cats, dogs, pigs, and non-human primates. Due to their small size, short lifespan, and rapid reproduction rate, mouse and rat models are particularly prevalent [63]. This review focuses on four model types: chemical-induced, genetic, diet-induced, and neovascularization models.

#### 4.2.1. Chemical-Induced Models

Chemical-induced models, which are known for their simple development and distinctive pathological changes, are prevalently utilized in DR research. Initially, alloxan was utilized to induce diabetes: this uric acid derivative selectively impairs pancreatic β-cells, and the death of β-cells leads to insulin release and subsequent hypoglycemia, with diabetes onset occurring within 24 h [64]. However, STZ has superseded alloxan in recent years due to its superior stability and rapid disease induction, thus becoming the gold-standard drug for diabetic research [65]. STZ acts mainly through the destruction of pancreatic islets and the ablation of islet β-cells in Langerhans [66]. Elevated blood glucose levels can be observed at one month after STZ injection, and this hyperglycemic state can be sustained for 22 to 24 months [67]. Early pathological changes, such as BRB disruption, thinning of the retinal inner nuclear layer and outer nuclear layer, reduction of neuronal cell numbers, and increased neuroglial cell apoptosis, appear approximately one month after STZ injection. Subsequent microvascular changes include increased permeability, neovascularization, and capillary basement membrane thickening [68,69]. Additionally, retinal dysfunction can be observed, such as increased retinal oxidative stress markers, pro-inflammatory factor levels, and pro-angiogenic markers [70,71,72]. In STZ-induced rat and mouse models, retinal morphological changes are almost identical; however, there are some differences in drug dose and disease progression [73].

#### 4.2.2. Genetic Models

The *Ins2^Akita^* mouse serves as a typical type 1 diabetes model. In this model, the *Insulin 2* gene is mutated, leading to a progressive loss of function in pancreatic islet β-cells. *Ins2^Akita^* mice develop pronounced hyperglycemia by four weeks. By 12 weeks, DR manifests, characterized by increased vascular permeability and reactive microglia. At 22 weeks post-hyperglycemia onset, there is a decrease in the thickness of the inner plexiform and inner nuclear layers of the retina, accompanied by a reduction in the number of retinal ganglion cells [74].

The NOD mouse is another commonly used type 1 diabetes model, where pancreatic islet β-cells are destroyed by CD4+ and CD8+ T cells through an autoimmune response [75]. Hyperglycemia typically initiates at 12 weeks. Four weeks after the onset of hyperglycemia, increased cell apoptosis, vascular abnormalities, and elevated VEGF levels can be detected in NOD mice [76].

The *db/db* mouse model, which is a spontaneous mutant strain, is a typical type 2 DM model characterized by leptin receptor mutation, which induces obesity and hyperglycemia. Additionally, a decreased number of RGCs and an increased thickness of the central retina can be observed as early as 6 weeks, thus progressing to BRB disruption, pericyte loss, RGC apoptosis, glial cell activation, and increased levels of VEGF, oxidative stress markers, and pro-inflammatory cytokines by 15 months [77]. The *db/db* mouse model is considered a reliable model for studying diabetes-induced optic neuropathy and for exploring the potential capacities of therapeutic agents in DR [78].

The *ob/ob* mouse model serves as another typical type 2 diabetes model, characterized by a mutation in the gene encoding the obese protein, which leads to leptin deficiency. This deficiency prompts hepatic lipogenesis and gluconeogenesis, while hyperglycemia stimulates insulin secretion, creating a negative cycle of insulin resistance. Hyperglycemia is observed at three weeks, and electroretinography reveals early retinal function loss by six weeks. By 20 weeks, a significant reduction in retinal thickness and the number of nuclei in the inner retinal layer are noted. Compared to the *Ins2^Akita^* and NOS models, DR onset occurs at approximately 8–12 weeks in the *ob/ob* mouse model, making it suitable for early DR research [79].

#### 4.2.3. Diet-Induced Models

Diet-induced models typically involve feeding rodents a high-fat diet to induce insulin resistance. This induction may occur alone or in combination with STZ. Rodents are usually induced with STZ either at the onset of the high-fat diet or 2–8 weeks after feeding on the diet, which reduces the amount of STZ required and thus minimizes its damage to other tissues [80]. However, diet-induced models are usually characterized by a lengthy development time and high experimental costs.

#### 4.2.4. Neovascularization Models

Retinal neovascularization is a major factor contributing to blindness in DR, and the establishment of related animal models is critical for studying the disease mechanism. The oxygen-induced retinopathy (OIR) model is the most commonly used animal model for investigating retinal neovascularization diseases. It offers the advantages of simple construction and high reproducibility.

In the OIR mouse model, mice and their lactating dams are placed in an environment of 75% ± 2% oxygen for five days, starting on day seven. Subsequently, the mice are returned to a standard oxygen environment with a concentration of 21% [81]. The rat model follows a similar protocol, and neovascularization is observed upon return to the normal oxygen environment [26]. The OIR model is characterized by two developmental phases: the immature vascular occlusion phase and the neovascularization pathologic proliferation phase.

**Figure 3:**
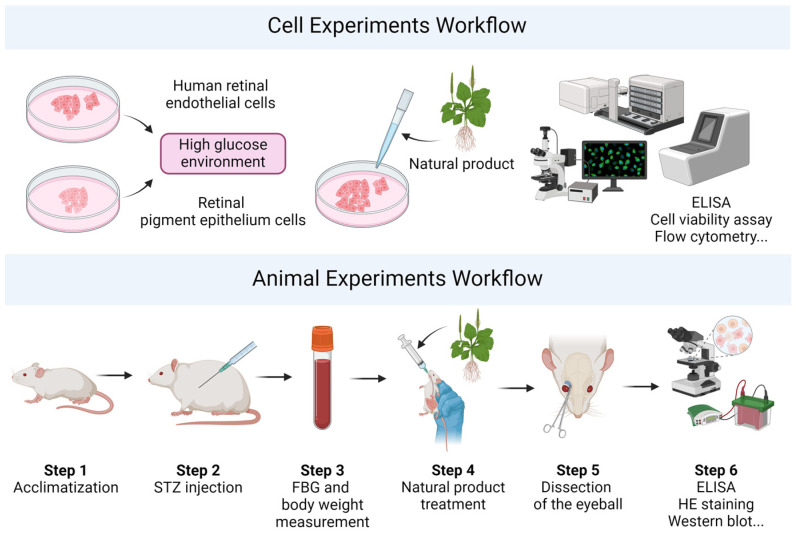
Workflow of cell and animal experiments. In vitro, cells are initially established in a high-glucose environment to simulate hyperglycemic conditions. Subsequently, cells are cultivated with natural product extracts. A series of experiments are then conducted to detect alterations in intracellular markers. In vivo, the experiment begins with the acclimatization of animals to a new environment. Following this, a calculated dose of streptozotocin (STZ) is administered. Throughout the experiment, fasting blood glucose (FBG) and body weight of animals are monitored. Oral treatment with natural product extracts is then administered over a period. Subsequently, the animals are euthanized, and the eyeballs are dissected. Finally, the therapeutic effects of natural products in diabetic retinopathy (DR) are validated through a series of experiments.

Cell and animal experiment workflows are shown in Figure 3. It is well known that cell and animal models offer predictive insights into disease mechanisms and potential therapeutic benefits of drugs. However, due to the complexity and heterogeneity inherent in human pathophysiology, these models may not fully simulate disease progression within the human body [64]. Despite this limitation, such experimental models are foundational to clinical research, thus emphasizing the importance of selecting appropriate models for research. Moreover, the translation of these findings to clinical practice necessitates rigorous validation to confirm drug efficacy in patients.

## 5. Natural Products in the Treatment of Diabetic Retinopathy

**Figure 4:**
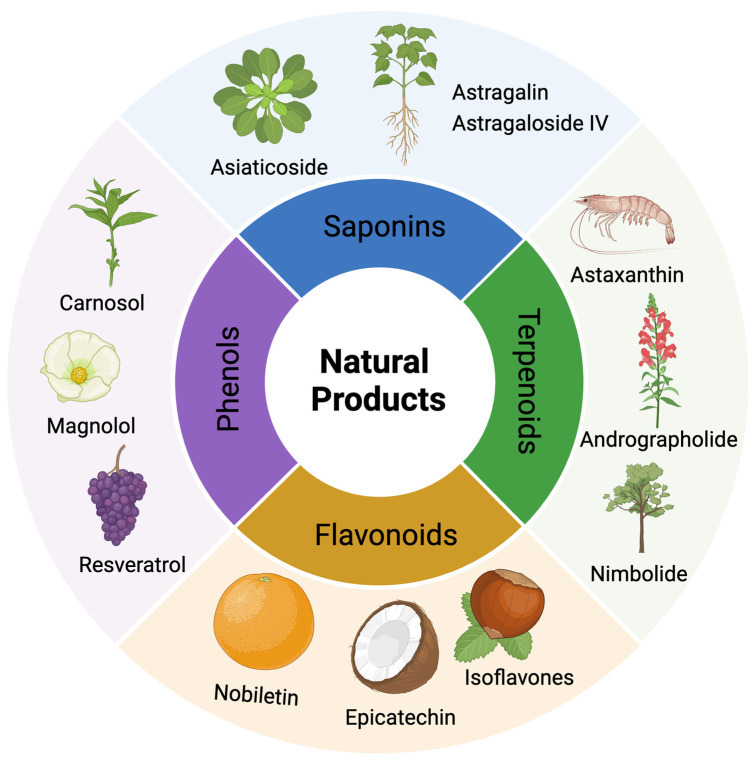
Classifications and natural sources of natural products.

**Figure 5:**
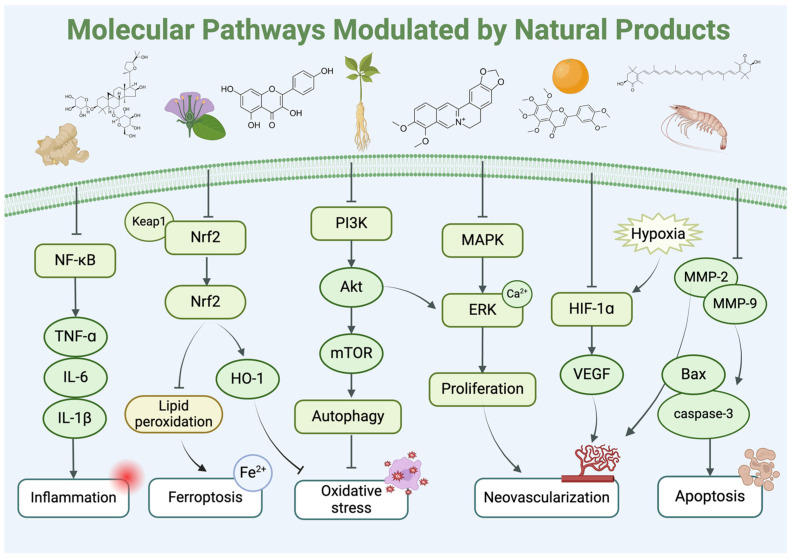
Molecular pathways modulated by natural products in diabetic retinopathy. Various natural products play regulatory roles in inflammation, ferroptosis, oxidative stress, neovascularization, and apoptosis-related signaling pathways in diabetic retinopathy. Abbreviations: NF-κB, nuclear factor kappa-B; TNF-α, tumor necrosis factor-alpha; IL-6, interleukin 6; Keap1, Kelch-like ECH-associated protein 1; Nrf2, nuclear factor erythroid-2-related factor 2; HO-1, heme oxygenase-1; PI3K, phosphoinositide 3-kinase; mTOR, mammalian target of rapamycin; MAPK, mitogen-activated protein kinases; ERK, extracellular signal-regulated kinase; HIF-1α, hypoxia-inducible factor-1 alpha; VEGF, vascular endothelial growth factor; MMP 2, matrix metalloproteinase 2.

The use of NPs and their metabolites is expanding in the therapeutic management of various diseases. Currently, there is increasing interest in natural extracts as alternative treatment approaches for DR [82,83]. Each category of NPs is discussed separately with respect to their protective role in DR (Table 1 and Table 2; Figure 4 and Figure 5).

### 5.1. Key Natural Products in DR Treatment

#### 5.1.1. Astragaloside-IV

Astragaloside-IV (AS-IV) is the main bioactive agent of *Astragalus membranaceus* and has excellent antioxidant capacity. Tang et al. [55] found that high-glucose conditions promoted ferroptosis in RPE cells. Moreover, AS-IV inhibited miR-138-5p expression, augmented Sirt1/Nrf2 pathway activity, and enhanced cellular antioxidant defense, thus leading to a reduction in ferroptosis in RPE cells. Ting et al. [84] noted that AS-IV significantly repressed aldose reductase (AR) activation, NF-κB overexpression, and ERK1/2 phosphorylation in a *db/db* mouse model, thus exerting anti-inflammatory and neuroprotective effects. Additionally, AS-IV was found to decrease apoptosis and dysfunction in RGCs. Zhao et al. [85] reported that AS-IV administration increased retinal thickness, alleviated DR-induced histopathological changes, and moderated elevated blood glucose levels in diabetic rats. AS-IV may exert an anti-inflammatory effect through the PI3K/AKT pathway.

#### 5.1.2. Resveratrol

Resveratrol is a polyphenolic compound that has been shown to have favorable ROS-scavenging efficacy across multiple pathways. In age-related macular degeneration, some studies have demonstrated that resveratrol has a beneficial impact on the retina [116,117]. In the STZ-induced diabetic rat model, resveratrol diminished the inflammatory response and retinal damage through the PON1 pathway, thus elevating PON1 expression while reducing Ox-DLD, advanced glycosylated end products (AGEs), and inflammatory factors (IL-1β, IL-6, TNF-α, Interferon-γ, MCP-1, and VEGF). Additionally, resveratrol reduced caspase-3 activity and Ox-LDL expression in rat retinal endothelial cells [86]. Hussaini found that resveratrol regulated the expression of multiple genes and proteins related to apoptotic pathways in RPE cells. It was observed that the transcript levels of the pro-apoptotic genes, *tumor suppressor protein 53* (*p53*), *BAX*, *caspase-3*, and *caspase-9*, and the anti-apoptotic gene *Bcl-2*, were increased, which consequently led to G2/M arrest and thus reduced apoptosis. Therefore, one of the protective pathways of resveratrol against diabetes-induced damage in RPE cells was through the inhibition of endogenous apoptotic pathway-related proteins [87]. Soufi found that long-term treatment with resveratrol reduced hyperglycemia and HbA1c levels, while improving energy metabolism and attenuating weight loss. Additionally, four months of oral resveratrol treatment decreased retinal oxidative stress and NF-κB activity, thereby reducing apoptosis rates in the retina of diabetic rats [88]. Ca^2+^/calmodulin-dependent protein kinase II (CaMKII), a serine-threonine protein kinase involved in neuronal cell death, was also found to be effectively inhibited by resveratrol, which prevented diabetes-induced apoptosis and upregulated CaMKII expression in RGCs. Kim et al. hypothesized that resveratrol may modulate CaMKII expression by interfering with calcium- and voltage-gated calcium channel-regulated processes, ultimately exerting neuroprotective effects in DR [89]. Furthermore, some clinical trials have shown that resveratrol is well tolerated, has relatively few side effects, and there is no clear evidence of serious adverse events directly attributable to resveratrol [118]. In conclusion, resveratrol may be a promising drug for the treatment of DR.

#### 5.1.3. Astaxanthin

Astaxanthin (ASX), which is a natural compound prevalent in a variety of plants and animals, is known for its anticancer and antioxidant functions. In the STZ-induced diabetic rat model, ASX increased retinal thickness and ganglion cell numbers. Furthermore, ASX suppressed Keap1 expression, facilitated Nrf2 translocation into the nucleus, and enhanced the expression of heme oxygenase-1 (HO-1), quinone oxidoreductase 1 (NQO1), γ-glutamylcysteine synthetase, and glutathione (GSH) peroxidase (GPx) [40]. Additionally, ASX mitigated DR-related damage by decreasing AGEs production and reducing pericyte apoptosis and inflammation. Although ASX did not lower elevated blood glucose levels in a rat model, its anti-inflammatory, antioxidant, and antiapoptotic effects also contributed to attenuating DR pathology [90].

#### 5.1.4. Quercetin

Quercetin is a major representative of the flavonoid family that can be found in various vegetables and fruits. Quercetin plays an important role in treating numerous diseases by exerting anti-inflammatory, antioxidant, anti-neovascular, and neuroprotective effects [119,120,121]. In diabetic rats, quercetin supplementation increased neurotrophic factors and inhibited cytochrome c and caspase-3, thereby protecting the retina from oxidative stress and apoptosis. Hence, quercetin was deemed to protect retinal neurons against DR via the BDNF/TrkB/Akt/synaptophysin pathway [91]. Additionally, quercetin modulated the activities of SOD and CAT, increased GSH levels, and prevented the release of TNF-α and IL-1β, thus suppressing NF-kB and caspase-3 activity. It also prevents retinal edema by inhibiting glial fibrillary acidic protein and aquaporin-4 elevation [92].

#### 5.1.5. Berberine

Berberine (BBR), which is a bioactive alkaloid isolated from the traditional Chinese medicine *Rhizoma Coptidis* [122], is known for its extensive medical value, including its anticancer, antiviral, and antibacterial effects [123,124]. Insulin injection is one of the most important treatment modalities for patients with type 1 DM and advanced type 2 DM. However, some studies have shown that insulin treatment may accelerate the progression of DR [125]. Wang et al. [31] reported on the role of BBR in suppressing DR progression in insulin-treated type 1 and type 2 diabetic mice. Insulin treatment activated retinal endothelial cells, which led to increased expressions of HIF-1α and VEGF, whereas BBR reversed these effects by inhibiting the Akt/mTOR signaling pathway. BBR improved retinopathy and reduced neovascularization and neuronal injury, thus complementing the effect of insulin therapy. Tian et al. [93] found that BBR attenuated interleukin-induced damage to retinal endothelial cells and suppressed the expression of inflammatory and oxidative stress markers under hyperglycemic conditions, which is likely partially through inhibition of the NF-κB signaling pathway. To further investigate the core mechanism by which BBR ameliorates DR, Na et al. [94] used four-dimensional independent data acquisition proteomics combined with bioinformatics analysis and experimental validation. These approaches further confirmed the therapeutic potential of BBR and highlighted its regulatory influence on molecular networks, particularly considering the significance of carbonic anhydrase 1.

### 5.2. Other Potential Natural Products in DR Treatment

#### 5.2.1. Saponins

Asiaticoside, which is a major active compound isolated from *Centella asiatica*, is known for its anxiolytic, scar healing, analgesic, antibacterial, anti-inflammatory, and antioxidant effects [126,127]. In high-glucose-cultured RPE cells, asiaticoside reduced the levels of inflammatory cytokines, including TNF-α, IL-6, and IL-1β, and reversed the upregulated expression of apoptosis-related proteins. Moreover, the protective effects of asiaticoside were abolished when the cAMP inhibitor SQ22536 was introduced. Consequently, asiaticoside may ameliorate the inflammatory response and apoptosis in DR by activating the cAMP/PKA signaling pathway [95].

Astragalin is another bioactive compound that is produced by *Astragalus membranaceus* and has beneficial effects on hyperglycemia [128,129,130]. In Müller cells, astragalin significantly reduced VEGF expression, thereby diminishing retinal neovascularization [96].

#### 5.2.2. Phenols

Carnosol, which is a phenolic extract from the herb rosemary, is known for its antioxidant properties. In HRECs cultured under high-glucose conditions, carnosol at concentrations of 10 µM or 20 µM facilitated cellular rejuvenation. It also attenuated cell apoptosis and ROS accumulation. Consequently, carnosol activated Nrf2/HO-1 signaling via the modulation of the ERK1/2 pathway, thereby mitigating high-glucose-induced damage in HRECs [97].

Magnolol, which is extracted from Magnolia officinalis, has potent anti-inflammatory, antioxidative, and anticancer effects [131,132,133]. In high-glucose- or S100b-induced (a specific receptor for AGEs ligand) RPE cells, magnolol inhibited the ERK/MAPK/Akt signaling pathway, thereby preventing the upregulated expression of transforming growth factor-β1 (TGF-β1) and fibronectin. It also curtailed lipid peroxidation triggered by S100b, thus decelerating the pathologic development of DR [56].

#### 5.2.3. Terpenoids

Nimbolide, which is a terpenoid found in neem plants, has been reported to be advantageous for cancer therapy [134,135]. This research demonstrated that nimbolide decreased inflammatory markers, alleviated oxidative stress, enhanced antioxidant defenses, and suppressed the TLR4/NF-κB pathway in diabetic rats. Moreover, its effect was similar to that of metformin, thus demonstrating its potential as a complementary DR therapy [98].

*Cyclocarya paliurus* is a traditional Chinese herbal medicine, and arjunolic acid (AA) is the major active compound that has significant protective effects against a variety of metabolic diseases. AA attenuated weight loss and increased retinal thickness and nucleus numbers in diabetic rats. Moreover, the protein expression of HO-1 was upregulated. In addition, AA modulated autophagy via the AMPK/mTOR pathway, thereby attenuating retinal cell damage and apoptosis. This study demonstrated that AA could treat DR by attenuating retinal cell damage and reducing oxidative stress, inflammation, and apoptosis through the AMPK/mTOR/HO-1/autophagy pathway [37].

Palbinone (PB), which is a triterpenoid isolated from *Paeonia suffruticosa*, decreased the levels of pro-inflammatory cytokines (IL-18 and IL-1β) and enhanced the activities of antioxidant enzymes, including superoxide dismutase (SOD), catalase (CAT), and GSH peroxidase (GPx) in STZ-induced diabetic rats. Furthermore, PB activated the Nrf2 pathway, elevated HO-1 expression, and inhibited NLRP3 inflammasome formation, thereby attenuating retinal inflammation and oxidative stress in DR [99].

The natural compound andrographolide is derived from the medicinal herb *Andrographis paniculate Nees*. Overproduction of indoleamine 2,3-dioxygenase (IDO), which is a pivotal enzyme in the kynurenine pathway, leads to oxidative stress in DR [136]. Andrographolide notably curbed IDO production and consequential changes in kynurenine metabolites, thereby attenuating the oxidative stress level in DR. In addition, it reduced oxidative stress markers, thus suggesting that it is a promising antioxidative therapeutic agent [100].

Wang et al. [49] isolated six compounds from Salvia and elucidated their structures via spectroscopy and X-ray diffraction. In particular, glechomanamide B hindered VEGF-induced tube formation in HUVECs by affecting the VEGF2 signaling pathway, thus potentially inhibiting DR-related neovascularization.

Tanshinone IIA is sourced from the traditional Chinese herb *Salvia miltiorrhiza* and is known to have cardiovascular benefits [137,138]. In recent years, tanshinone IIA has been found to have specific effects on DR. The molecular mechanism, pharmacodynamic target, and protein interaction network of tanshinone IIA were analyzed by using integrated pharmacology and verified by animal experiments. Tanshinone IIA restored the retinal structure of the rats, decreased the mRNA expressions of VEGF, IL-1β, IL-6, TNF-α, and caspase-3, and modulated the protein expression of Bcl-2, Bax, and VEGFA. These results suggested that tanshinone IIA could alleviate hyperglycemia-induced retinal damage, thus offering anti-inflammatory and antiangiogenic benefits in DR [101].

#### 5.2.4. Flavonoids

Flavonoids are heterocyclic compounds that can be widely found in various vegetables and fruits, such as tomatoes, grapes, and nuts. Flavonoids are known for their significant antioxidant properties, which can potentially mitigate oxidative stress and neurodegeneration in DR [83].

Kaempferol is a medicinal flavonol that can be found in numerous fruits and vegetables. It has been shown to inhibit lactate dehydrogenase release, apoptosis, caspase-3 activity, and ROS levels in RGCs. Moreover, kaempferol potentially protected RGCs from retinopathy induced by high glucose by modulating the ERK/VASH1 signaling pathway [102]. In HRECs cultured under high-glucose conditions, kaempferol decreased VEGF and placental growth factor (PGF) levels and inhibited cell proliferation and migration. Kaempferol may inhibit the Src/Akt1/Erk1/2 signaling pathway, thus decreasing retinal neovascularization [103].

Tilianin (TN) is a natural polyphenolic flavonoid. Oral administration of TN to diabetic rats markedly decreased their food consumption, blood glucose level, and serum insulin status. TN increased the expression of nuclear factor erythroid-2-related factor 2 (Nrf2) and its target gene HO-1, while decreasing thioredoxin-interacting protein (TXNIP), NOD-like receptor protein 3 (NLRP3), apoptosis-associated speck-like protein containing a CARD, caspase-1, and IL-1β protein levels. TN thereby manifests potent antioxidant and anti-inflammatory effects in DR, potentially by modulating the Nrf2/TXNIP/NLRP3 inflammasome pathways [104].

Chrysin is a member of the flavonoid family, and the structure of chrysin can modulate the immune system [139]. In addition, chrysin notably decreased the phosphorylation of AKT, ERK, and MMP-2 in RF/6A cells cultured in a high-glucose environment, thus reducing the influence of VEGF and its receptor VEGFR, and thereby curbing RE/6A cell migration [50].

Morin is a dietary bioflavonoid from the Moraceae family of plants. Moreover, morin has been shown to reduce lipid peroxidase activity and enhance the levels of endogenous antioxidants (GPx, CAT, and SOD) in the retina. Further, morin can decrease the concentrations of TNF-α, IL-1β, and VEGF, and contribute to increases in retinal thickness and ganglion cell numbers. Thus, morin may attenuate retinal damage by exerting antioxidant effects in STZ-induced diabetic rat models [105].

Gambogic acid (GA) is a flavonoid natural product extracted from the traditional Chinese medicine gamboges resin. GA can suppress proliferation, migration, and tube formation in high-glucose-cultured RF/6A cells. GA can also ameliorate retinal morphology in diabetic mice by suppressing neovascularization via the inhibition of HIF-1α and VEGF. Furthermore, the PI3K/AKT signaling pathway has been implicated in the inhibitory effects of GA on DR [51].

DHF (7,8-dihydroxyflavone) is a natural extract from non-mammalian animals or plants, such as citrus plants, grains, tea, vegetables, and fruits. In high-glucose-injured RPE cells, DHF significantly reduced the expression of the apoptotic factor caspase-9 at both the RNA and protein levels. DHF also attenuated apoptosis by activating the TrkB signaling pathway, whereas the TrkB antagonist K252a inhibited the protective effect of DHF by dephosphorylating TrkB signaling [106].

Nobiletin is the major polymethoxylated flavone isolated from citrus fruits. Matrix metalloproteinases (MMPs) play a promoting role in the development of PDR; however, the clinical application of many MMP inhibitors is restricted due to their non-specificity, low bioavailability, and adverse effects. This study demonstrated that nobiletin can reduce the enzymatic activity of MMP-9 by inhibiting its gene transcription and by augmenting the production of tissue inhibitor of metalloproteinase-1 in Müller cells. Moreover, the metabolite of nobiletin, 4′-demethylated nobiletin, can ensure its efficacy in the body. A further understanding of the bioavailability of nobiletin is needed to assess its bioactivity within target tissues [107].

Isoflavones are a member of the flavonoid family that have strong antioxidant effects. In STZ-induced diabetic rats, isoflavones exerted potent antioxidative effects, thus reducing retinal sorbitol accumulation by decreasing AR enzyme activity, thereby slowing DR progression. Additionally, isoflavones decrease thiobarbituric acid-reactive substances and protein carbonyl levels in a dose-dependent manner while enhancing GSH, thus diminishing oxidative stress in the retina [108].

Epicatechin, which is a prevalent flavonoid found in human dietary sources, is recognized for its role in enhancing insulin responsiveness and managing blood glucose levels [140,141]. Increases in AGEs are strongly associated with diabetic retinopathy [142]. Epicatechin disrupted AGE antigen formation and mitigated AGE accumulation in a dose-dependent manner in the retina. Moreover, in diabetic rats exogenously injected with AGEs, epicatechin can also ameliorate retinal vascular cell apoptosis and reduce the AGEs burden [109].

#### 5.2.5. Saccharide

Fucoidan is a polysaccharide compound extracted from brown seaweeds that can be found in the sea and on land. In high-glucose-cultured RPE cells, fucoidan prevented ROS production by inhibiting the Ca^2+^-dependent ERK1/2 signaling pathway, thereby attenuating the retinal damage attributed to high glucose [110]. In addition, in high-glucose-induced microvascular endothelial cells, fucoidan inhibited vascular endothelial cell proliferation and VEGF expression, thus demonstrating antiangiogenic effects. In conclusion, fucoidan exerted similar effects as calcium dobesilate in alleviating retinal pathological changes and blocking neovascularization in a *db/db* type 1 diabetic mouse model [111].

#### 5.2.6. Quinone

Aloe-emodin (AE), a natural compound derived from the traditional Chinese medicine Radix et Rhizoma Rhei, has been known to possess antiangiogenic, antioxidant, and anti-tumor properties [143]. Wu et al. discovered that AE inhibited hypoxia-induced retinal neovascularization via the HIF-1α/VEGF signaling pathway, utilizing both an in vitro model of CoCl_2_-induced VEGF secretion and an in vivo model of oxygen-induced neovascularization. In vitro, the mRNA expressions of VEGFA and prolyl hydroxylase-2 (PHD-2) were downregulated, and the protein levels of VEGFA, HIF-1α, and PHD-2 decreased. In vivo, AE inhibited retinal neovascularization in the OIR rat model. This study suggests that AE may potentially treat DR by inhibiting retinal neovascularization [112].

#### 5.2.7. Steroid

*Trigonella foenum-graceum* Linn. (Fenugreek) is an annual herb that was initially used as a vegetable and spice in India. Fenugreek seeds contain various saponins, including 4-hydroxyisoleucine, trigonelline, and diosgenin. Gupta et al. discovered that fenugreek played a crucial role in attenuating oxidative stress and inhibiting inflammatory and angiogenic molecular biomarkers in diabetic rats. Additionally, fenugreek was found to inhibit basement membrane thickening and BRB destruction [144]. Diosgenin, an important natural compound extracted from fenugreek seeds, was found to enhance cell viability in RPE cells under high-glucose conditions, and reduce inflammation, oxidative stress, and apoptosis levels through the activation of the AMPK/Nrf2/HO-1 signaling pathway. Moreover, the protective effects of diosgenin on high-glucose-induced RPE cells were reversed by dorsomorphin (an AMPK inhibitor) [113]. Liao et al. demonstrated that diosgenin increased the total retinal thickness, photoreceptor layer, and outer nuclear layer thickness, and ameliorated ganglion cell loss in the *db/db* mouse model. Additionally, diosgenin was shown to increase Bcl-2 expression while reducing caspase-3 expression, suggesting its potential as an anti-apoptosis agent in vivo [114]. These studies suggest that fenugreek and its extracts may serve as a new modality for DR treatment.

#### 5.2.8. Vitamin D

Vitamin D is one of the essential nutrients for the body to maintain normal physiological functions, and it is extensively employed as a dietary supplement for health preservation [145]. Most of the studies have aimed to investigate whether individual vitamin D serum levels are associated with DR, yet the conclusions have remained inconclusive.

A cross-sectional study by Long et al. assessed the relationship between vitamin D deficiency and DR severity in 842 individuals stratified according to glycemic control, and they demonstrated that vitamin D deficiency may be associated with severe DR only in those individuals with well-controlled blood glucose levels [146].

The findings of Alcubierre et al. suggested that vitamin D deficiency may be associated with DR severity; however, vitamin D consumption did not significantly differ between DR patients and healthy individuals [147]. Moreover, a longitudinal investigation by Millen et al. over three years also did not observe a connection between vitamin D and DR [148].

In conclusion, vitamin D supplementation may be advantageous for treating DR, but additional large-scale randomized controlled trials are needed.

#### 5.2.9. Natural Products with Various Bioactive Agents

*Panax notoginseng saponins* (PNS), which are the primary constituents of *Panax notoginseng*, exhibit a variety of anti-inflammatory, antioxidant, and neuroprotective properties. Investigations have demonstrated that ginsenoside Rg1 (GRg1) and GRb1, which are key components of the PNS, exerted anti-inflammatory effects in DR. GRg1 and GRb1 increased the retinal inner nuclear layer thickness, decreased retinal acellular capillaries, alleviated BRB destruction, eliminated microglial activation, reversed leukocyte adhesion, and suppressed elevated inflammatory factor levels in the serum. In conclusion, GRg1 and GRb1 decreased retinal damage, attributable to high glucose through the NF-κB signaling pathway [14].

The deciduous plant *Ulmus davidiana*, which is extensively cultivated in China, South Korea, and many other countries, serves as a traditional herbal remedy because its roots and stems are used to alleviate numerous ailments, such as cancer and asthma [149,150,151,152]. The primary active bioactive compounds that have been identified in *Ulmus davidiana* are a 60% edible ethanolic extract (U60E) and the catechin 7-O-β-D-apiofuranoside (C7A). In high-glucose- and TNF-α-stimulated cells, U60E and C7A inhibited pericyte apoptosis by suppressing p38 and JNK activities. Additionally, when pericytes and endothelial cells were co-cultured, U60E and C7A were found to restore the suppressed expression of connexin ZO-1 and reduce the increased vascular permeability [115].

## 6. Mechanisms of Action, Toxicity, and Drug–Drug Interactions of Natural Products

### 6.1. Bioavailability and Ocular Metabolic Pathways of Natural Products

The main methods of drug delivery into the ocular system include intravitreal injections and systemic, periocular, subretinal, suprachoroidal, or topical administration [153]. Specifically, for DR, the inhibition of neovascularization is often achieved through the intravitreal injection of VEGF antibodies. NP supplements offer the unique advantage of efficiently reaching the retina through oral systemic administration. Within the body, NPs are metabolized, assimilated into the bloodstream, traverse the BRB, and subsequently nourish retinal cells. RPE cells have the capacity to take up vitamins and nutrients from the blood circulation and deliver them to RGCs [154].

However, some current NPs are limited by their low efficiency in drug delivery. Therefore, more research has focused on improving drug delivery systems through nanotechnology. Nano-synthetic drugs potentially improve the safety of systemic delivery and ensure drug targeting. Currently, an increasing number of new nanomaterials are emerging for DR treatment, thus offering non-invasive and safe drug delivery or modification strategies [155]. Although quercetin is a promising therapeutic drug for DR, its high oral dose requirement and limited bioavailability have limited its clinical use [156,157,158]. Gui et al. reported that ultrasmall Fe-Quer nanozymes (NZs), which are synthesized by binding quercetin with low-toxicity ions, showed increased delivery efficacy, robust ROS scavenging, and vascular protection in both in vivo and in vitro experiments. Histologic examination further confirmed the low toxicity of Fe-Quer NZs, thus suggesting a safe and effective drug delivery system [159]. Moreover, Toragall et al. designed a lutein-loaded chitosan-sodium alginate nanocarrier comprising an oleic acid core (LNC) for improving the solubility, instability, and bioavailability of lutein. Studies have indicated that LNCs with a smaller size and smooth spherical morphology do not affect normal cell viability at a concentration of 20 μM, and LNCs exhibit a high cellular uptake rate, even under H_2_O_2_-induced oxidative stress conditions [160]. These observations provide avenues for the use of nanomaterials encapsulating NPs as a new therapeutic strategy for DR management.

Ocular drug clearance mainly occurs through the anterior chamber with aqueous humor circulation or through the BRB, with subsequent systemic circulation to the liver. The efficiency of ocular drug clearance depends on drug permeability, molecular size, and lipophilicity. Low-molecular-weight drugs permeate the BRB more efficiently than high-molecular-weight drugs [161], and protein clearance mainly occurs through BRB metabolism [162]. Furthermore, hydrophilic drugs tend to be primarily cleared by passive diffusion through the anterior chamber pathway, whereas lipophilic drugs are mainly cleared through the BRB. The progression of ocular diseases may induce changes in the pathophysiological environment of the eyes, which can correspondingly influence the drug’s metabolic profile [153]. Inappropriate metabolic processing of drugs may cause adverse reactions.

### 6.2. Toxicity and Side Effects

Safety remains the foremost concern in the utilization of pharmaceuticals. Currently, there has been a growing interest in natural compounds, some of which have been formulated as dietary supplements. NPs are generally considered to have lower toxicity and fewer side effects; therefore, they are currently widely used. However, rigorous clinical trial validation of the safety of many dietary supplements or herbal medicines is lacking, and indiscriminate supplementation with NPs may be harmful.

Many NPs are administered orally; additionally, due to their natural connotations, their potential toxicity may be underestimated. The significance of dosage for medical efficacy is well established. Overconsumption can disrupt normal metabolic pathways, thus leading to drug accumulation in certain organs and resulting in cytotoxicity [163,164]. Furthermore, drugs may be harmful due to overdose, metabolic processes, and drug-drug interactions (DDIs). For example, although moderate vitamin D supplementation helps to improve the body’s absorption of calcium and phosphorus, excessive supplementation can induce toxic side effects, such as hypercalcemia, vomiting, and dehydration [165,166,167].

The liver and kidneys serve as pivotal organs for metabolic processes, thus necessitating vigilant monitoring for potential hepatoxicity and nephrotoxicity when administering medications. BBR may suppress hepatic gluconeogenesis and potentially induce hepatoxicity by inhibiting energy metabolism and pyruvate carboxylation [168]. Pyrrolizidine alkaloids, which are toxic compounds found in numerous Chinese medicinal herbs and a small percentage of flowering plants, are known to cause hepatoxicity via metabolic activation by CYP450s to form toxic intermediates in the liver [169]. Teucirum chamaedrys is a traditional food and medicinal plant that has also been reported to have hepatoxicity [170]. In Latin America, there is an increasing incidence of hepatoxicity due to the use of herbal medicines and dietary supplements, and these patients have a higher rate of mortality or need for liver transplantation than those treated with traditional medications [171].

In this review, NPs did not show notable toxic effects in either in vivo or in vitro experiments. For example, polysaccharide compounds from algae were shown to be non-toxic at concentrations ranging from 100 μg/mL to the minimal effective dose of 0.1 μg/mL [172]. Furthermore, a meta-analysis by Li et al. reported of the safety of Chinese herbal compounds for the treatment of DR, with a significantly lower probability of adverse risks than in the conventional treatment group [173]. However, cellular and animal models may not fully predict human toxicological outcomes. Therefore, more research is needed to explore the possible adverse effects of NPs on the human body and provide more evidence for the future application of NPs.

### 6.3. Drug–Drug Interactions

DDIs are combined effects that manifest when multiple drugs are administered consecutively. These effects may enhance or diminish drug efficacy and, in some cases, lead to toxicity. DDIs are more likely to occur in those who suffer from chronic diseases or those undergoing treatment with a combination of traditional medicines and NPs [174]. Currently, NPs are often used in combination with conventional medicines, which certainly raises concerns regarding the potential for toxicity due to DDIs [175]. Studies have indicated that NPs can exert bidirectional influences on pharmacological agents; for example, BBR displays differential modulatory effects on the uptake of nimodipine by cerebral microvascular endothelial cells, with an inhibitory effect at high concentrations and an enhancing effect at low concentrations [176]. Enzymes involved in metabolism and transport proteins are also instrumental in the modulation of DDIs [177]. Co-administration of multiple compounds can cause competitive metabolic processes, which may alter drug bioavailability and pharmacokinetics, thus modifying therapeutic outcomes and possibly inducing adverse reactions [178,179]. For example, the co-administration of metformin and BBR has been shown to promote drug accumulation in hepatic and renal tissues, which is attributed to inhibited multidrug and toxin extrusion-1-mediated urinary and biliary excretion [180].

DDIs may also enhance therapeutic outcomes. Oh et al. explored the antioxidant effects of ascorbic acid in conjunction with ASX in RPE cells subjected to oxidative stress via H_2_O_2_ or UVB exposure. The results indicated an increase in cell viability and a concomitant decrease in intracellular ROS levels. Ultimately, the combination of ascorbic acid and ASX has been shown to amplify antioxidant effects [181]. In traditional Chinese medicine (TCM), several herbs are usually mixed in specific proportions to form a single formula, in which the therapeutic effects may be attributed to the synergistic, additive, and antagonistic effects of several different ingredients, thus ultimately enhancing its efficacy and reducing its toxicity. For example, LDD, which is a classical TCM formula, consists of six herbs. Its efficacy in DR treatment may be attributable to the synergistic effects of multiple active ingredients engaging in different biological targets and pathways [182]. Furthermore, a meta-analysis by Li et al., comparing the effects of combining traditional Chinese medicine and conventional Western medicine treatment for DR, showed that combining therapies could lower blood glucose levels, improve fundus hemorrhage, and restore visual function in DR patients [183].

## 7. Limitations and Perspectives

It is generally acknowledged that cell cultures and animal models cannot entirely mimic the entire process of disease progression. Any new therapeutic intervention must be based on rigorous basic research and clinical trials to determine the exact mechanisms and targets of the drug and validate its efficacy in large population cohorts. Most of the studies that were included in this review involved only cell and/or animal experiments, thus indicating the need for additional preclinical and clinical trials to confirm the effectiveness of these methods in populations. Additionally, it is critical to investigate whether the process of absorption, distribution, metabolism, and excretion of NPs in the human body will potentially cause toxicity or adverse effects and how to avoid the risks associated with the use and overdose of multiple drugs. NPs may interact through myriad mechanisms and targets, the complete mechanisms of which remain to be fully understood. By leveraging advancements in technology and bioinformatics, such as high-throughput screening, high-content screening, and virtual screening, we hope to facilitate our understanding of the definitive action of NPs.

## 8. Conclusions

DR is one of the most severe complications of DM, and its pathological changes include inflammation, retinal micro-vasculopathy, oxidative stress, and retinal neurodegeneration. The complexity of the multiple mechanisms underlying these pathologic changes in DR makes achieving satisfactory outcomes with current treatments challenging. Considering the pivotal role of dietary and lifestyle habits in diabetes management, therapeutic strategies based on diet present a promising frontier for DR treatment.

We noted that research predominantly concentrates on preclinical studies involving supplementation with one or more natural compounds that are administered either through cell culture or animal diets. In vitro studies usually employ cellular models, such as endothelial cells and RPE cells, whereas in vivo experiments frequently utilize STZ-induced diabetic rats or mice, as well as *db/db* mouse models.

This review summarizes current investigations on the potential impacts of NPs on DR. NPs still face some challenges, such as poor bioavailability, rapid breakdown, low targeting, and inconsistent distribution within the human body [184]. They are widely available and easily accessible, thus presenting advantages over small-molecule drugs. Moreover, NPs have demonstrated anti-inflammatory, antioxidative, antiangiogenic, and neuroprotective effects in diabetic models, and the associated signaling pathways include NF-kB, Nrf2/Keap1, cAMP/PKA, AMPK/mTOR/HO-1, and miR-138-5p/Sirt1/Nrf2. Future research incorporating nanomaterial-encapsulated NPs and employing bioinformatics-based NPs may lead to significant advances.

In conclusion, NPs are a non-invasive treatment with high therapeutic potential and are expected to be used either alone or in combination with other traditional Western medications in the future for the prevention of DR. We anticipate more preclinical and clinical research to validate the effects of NPs on DR.

## Figures and Tables

**Table 1:** Current research on the protective effects and mechanisms of natural products in DR.

Category	Compound Name	Natural Product	Cell/ Animal Model	Efficacy	Mechanism	References
Key Natural Products	Astragaloside-IV 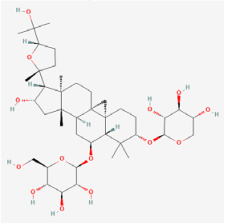	*Astragalus mambranaceus*	Human retinal pigment epithelium cells (ARPE-19 cells)/*db/db* mouse/STZ rats	1. Decreased the rate of ferroptosis by inhibiting expression of miR-138-5p 2. Decreased GSH content, mitochondria size, and ridge 3. Increased expression of glutathione peroxidase 4, glutamate cysteine ligase, and glutamate cysteine ligase catalytic subunit 4. Inhibited aldose reductase (AR) activation and the overexpression of NF-kB and ERK1/2 phosphorylation 5. Improved the amplitude in pattern electroretinogram and reduced apoptosis 6. Increased retinal thickness, alleviated DR-induced histopathological changes, and elevated blood glucose levels 7. Elevated DR-depressed protein levels of PI3K and AKT	1. miR-138-5p/Sirt1/Nrf2 2. PI3K/Akt	[55,84,85]
	Resveratrol 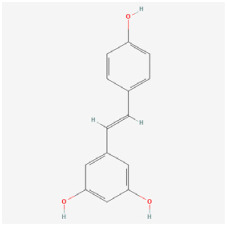	Grape, peanuts, berries	Rat retinal endothelial cells (RRECs)/STZ rats/STZ mice	1. Revocered the insulin level and PON1 expression and activity 2. Regulated retinal vascular permeability and mRNA expression of paraoxonase 1 (PON1), VEGF, bFGF, low-density lipoprotein (LDL), and high-density lipoprotein (HDL) 3. Inhibited retinal apoptosis by regulating Ox-LDL, caspase-3, and PON1 activity 4. Reduced inflammatory factors of IL-1β, IL-6, TNF-α, VEGF, Interferon-γ, and MCP-1 5. Mediated alterations in gene and protein expressions related to apoptosis 6. Enhanced SOD activity and reduced the 8-isoprostane level and GSSG/GSH ratio 7. Improved retinal layer disorganization and attenuated retinal thickness reduction 8. Blocked the increase in CaMKII and phosphor-CaMKII protein levels	1. PON1 2. NF-κB	[86,87,88,89]
	Astaxanthin (ASX) 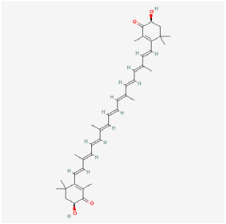	*Haematococcus pluvialis*, shrimps	STZ rats	1. Alleviated the decrease in retinal thickness and ganglion cell layer cell loss 2. Downregulated inflammatory factors of TNF-α, IL-1β, IL-6, MIC-1, and IL-10 3. Exerted antioxidant effects by regulating GSH, GPx, and total antioxidant capacity, malonic dialdehyde (MDA), and ROS levels 4. Fostered the expressions of HO-1 and NQO1 5. Exerted anti-apoptosis effects by regulating caspase-3, Bcl-2, and BAX expressions 6. Decreased the endothelial cell/pericyte ratio and the number of acellular capillaries	Nrf2/Keap 1	[40,90]
	Quercetin 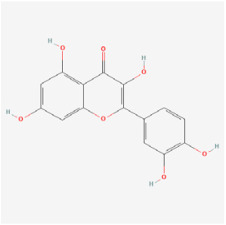	Licorice	STZ rats	1. Decreased the expression of BDNF, NGF, TrkB receptor, synaptophysin, and p-Akt 2. Attenuated apoptosis by decreasing caspase-3 and cytochrome c levels, and increasing Bcl-2 expression 3. Increased GSH levels 4. Decreased pro-inflammatory cytokines of TNF-α and IL-1β 5. Increased retinal thickness and cell count in the ganglion cell layer	BNDF/TrkB/Akt/synaptophysin	[91,92]
	Berberine 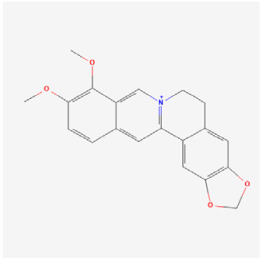	*Rhizoma Coptidis*	HRECs/STZ mice/STZ rats/*db/db* mice	1. Increased retinal layer thickness 2. Suppressed VEGF and HIF-1α expressions 3. Suppressed neovascularization 4. Reduced oxidative stress 5. Inhibited leukocyte-mediated killing of HRECs	1. Akt/mTOR/HIF-1α/VEGF 2. NF-κB	[31,93,94]
Saponins	Asiaticoside 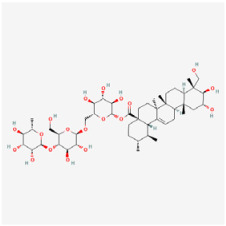	*Centella asiatica*	ARPE-19 cells	1. Restored the cell survival rate 2. Suppressed inflammation cytokines (TNF-α, IL-1β, and IL-6) 3. Regulated apoptosis levels (Bcl-2, Bax, caspase-3, and caspase-9) 4. Activated cAMP and PKA	cAMP/PKA	[95]
	Astragalin 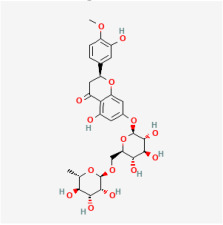	*Astragalus mambranaceus*	Rat retinal Müller cells	1. Decreased VEGF expression	VEGF	[96]
Phenols	Carnosol 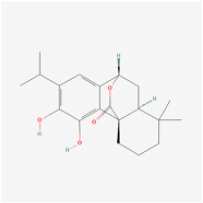	Rosemary	Human retinal endothelial cells (HRECs)	1. Increased cell viability and attenuated apoptosis 2. Induced HO-1 expression via Nrf2 activation	ERK1/2/Nrf2/HO-1	[97]
	Magnolol 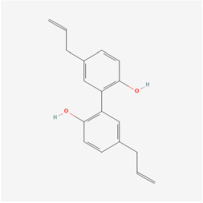	*Magoolia officinalis*	ARPE-19 cells	1. Prevented TGF-β1 and fibronectin expression 2. Inhibited ERK/MAPK/Akt activity	ERK/MAPK/Akt	[56]
Terpenoids	Nimbolide 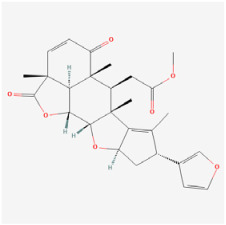	Neem plants	STZ rats	1. Suppressed inflammatory factors (TNF-α, IL-1β, and IL-6) 2. Suppressed MCP-1, VEGF, and MMP-9 levels 3. Improved antioxidant levels (SOD, GSH, SOD/CAT, and GSH/GSSG) 4. Suppressed TLR4 and NF-kB expressions	TLR4/NF-kB	[98]
	Arjunolic acid 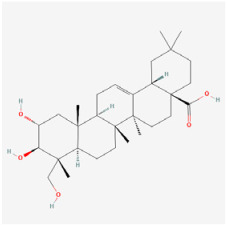	*Cyclocarya paliurus*	ARPE-19 cells/STZ rats	1. Prevented weight loss and increased the retinal thickness and nuclei counts 2. Inhibited oxidative stress, inflammation, and apoptosis 3. Upregulated the HO-1 protein level 4. Activated the AMPK/mTOR/HO-1/autophagy pathway	AMPK/mTOR/HO-1/autophagy	[37]
	Palbinone 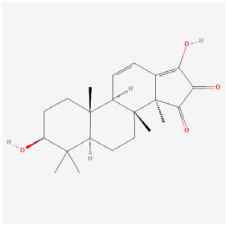	*Paeonia suffruticosa*	STZ rats	1. Improved morphometric and pathological changes of retina tissues 2. Enhanced antioxidant activities (SOD, catalase, and GPx) 3. Alleviated vascular permeability via inhibiting NLRP3 inflammasome formation 4. Reduced pro-inflammatory cytokines (IL-18, and IL-1β)	Nrf2/HO-1	[99]
	Andrographolide 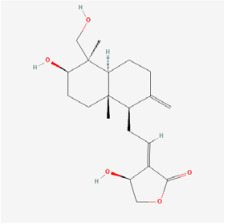	*Andrographis paniculate (Burm. F.) Nees*	STZ rats	1. Halted the sustained hyperglycemia and reversed the body weight and blood glucose level 2. Boosted cellular antioxidant defense by restoring the GSH level 3. Inhibited IDO and consequential changes in Kynurenine metabolites	-	[100]
	Glechomanamide B 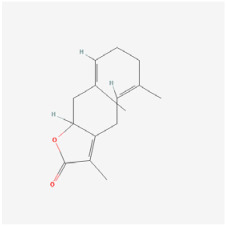	Salvia	Human microvascular endothelial cells (HMVECs)	1. Decreased the expressions of VEGF-R2, GLUT1, HK2, and ANGPT2 2. Inhibited tube formation induced by VEGF and BMP4	VEGF	[49]
	Tanshinone IIA 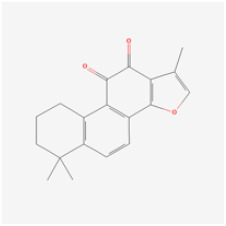	*Salvia miltiorrhiza* bunge	STZ rats	1. Exerted anti-inflammatory and antioxidation effects by regulating IL-1β, IL-6, TNF-α, SOD, GSH-PX, and MDA expressions 2. Regulated cell proliferation, apoptosis, and neovascularization	-	[101]
Flavonoids	Kaempferol 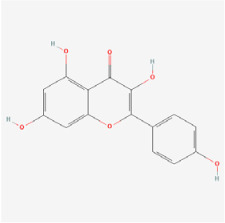	*Kaempferia galanga* L.	Retinal ganglion cells (RGCs)/HRECs	1. Attenuated apoptosis, caspase-3 activity, and LDH leakage 2. Promoted cell viability and decreased ROS levels 3. Inhibited cell proliferation, migration, and tube formation 4. Suppressed the expression of VEGF and PGF 5. Suppressed PI3L expression and ERK1/2, Src, and Akt1 activation	1. ERK/VASH1 2. Src/Akt1/ERK1/2	[102,103]
	Tilianin 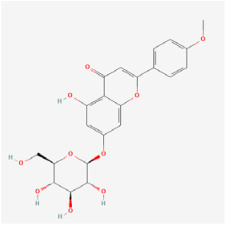	*Traversia baccharoides Hook.f.*	STZ rats	1. Reduced fasting glucose status, HbA1c levels, and augmented serum insulin status 2. Increased Nrf2 and HO-1 expressions and decreased MDA levels 3. Increased SOD, CAT, and GPx levels 4. Decreased expression of TXNIP, NLRP3, ASC, caspase-1, and IL-1β 5. Ameliorated retinal morphological and morphometric changes	Nrf2/TXNIP/NLRP3	[104]
	Chrysin 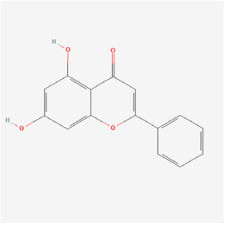	*Oroxylum indicum (Linn.) Bentham ex Kurz*	Choroid endothelial cells (RF/6A cells)	1. Inhibited cell migration via inhibiting AKT and ERK phosphorylation 2. Decreased MMP-2 expression 3. Inhibited HIF-1α and VEGF expressions	VEGF	[50]
	Morin 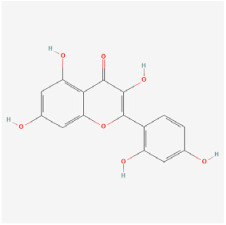	Moraceae family plants	STZ rats	1. Decreased blood glucose levels 2. Improved endogenous antioxidant enzymes’ activity (GPx, CAT, and SOD) 3. Reduced TNF-α, IL-β, and VEGF expressions 4. Increased retina thickness and cell count in the ganglion cell layer	-	[105]
	Gambogic acid 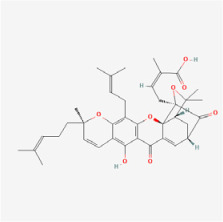	Gamboges resin	RF/6A cells/STZ mice	1. Suppressed cell proliferation, migration, and tube formation 2. Decreased HIF-1α and VEGF expressions 3. Attenuated retinal neovascularization	1. HIF-1α/VEGF 2. PI3K/Akt	[51]
	7,8-Dihydroxyflavone 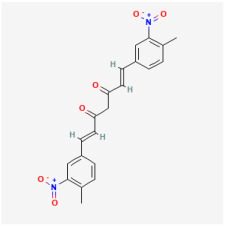	Citrus, grains, tea	ARPE-19 cells	1. Ameliorated apoptosis by inhibiting caspase-9 activity and phosphorylating TrkB protein	TrkB	[106]
	Nobiletin 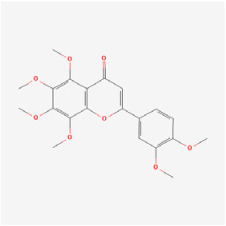	Citrus plants	Human retinal Müller cells (MIO-M1 cells)	1. Attenuated MMP-9 enzymatic activity 2. Regulated MMP-9 and tissue inhibitor of metalloproteinase-1 expression	PI3K/Akt	[107]
	Isoflavones 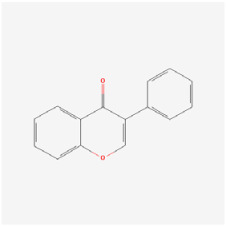	*Caesalpinia pulcherrima*	STZ rats	1. Inhibited AR activity 2. Decreased thiobarbituric acid-reactive substances and protein carbonyl levels 3. Restored GSH levels 4. Increased antioxidant enzymes’ activities (SOD, GPx, and catalase)	-	[108]
	Epicatechin 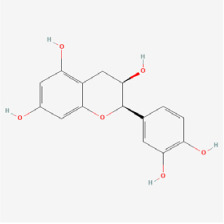	Green tea, coconut	AGE-injected rats/glycated bovine serum albumin	1. Reduced AGEs accumulation 2. Inhibited retinal apoptosis	-	[109]
Saccharides	Fucoidan 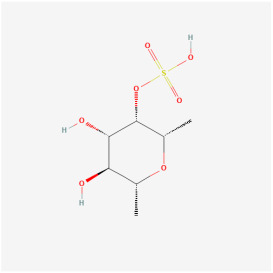	Brown algae	ARPE-19 cells/brain microvascular endothelial cells/STZ mice	1. Inhibited apoptosis and ROS generation 2. Inhibited high-glucose-mediated Ca^2+^ influx 3. Inhibited ERK phosphorylation 4. Inhibited cell proliferation, angiogenic vessels, and VEGF expression 5. Inhibited HIF-1α expression	1. Ca^2+^-dependent ERK 2. HIF-1α/VEGF	[110,111]
Quinones	Aloe-emodin 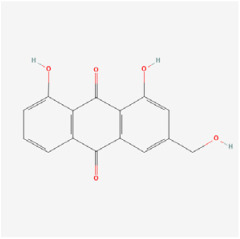	Radix et Rhizoma Rhei	ARPE-19 cells/OIR rats	1. Inhibited the secretion of VEGFA in the ARPE-19 cells under hypoxia conditions 2. Decreased the mRNA and protein expressions of VEGFA and PHD-2 in the ARPE-19 cells 3. Inhibited hypoxia-induced retinal neovascularization in vivo	HIF-a/VEGF	[112]
Steroids	Diosgenin 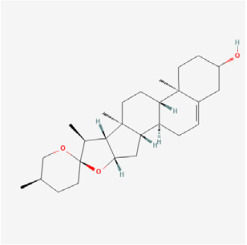	Fenugreek seeds, Wild yam roots	ARPE-19 cells/*db/db* mice	1. Increased the viability of ARPE-19 cells 2. Inhibited apoptosis of ARPE-19 cells 3. Reduced the inflammatory response and oxidative stress of ARPE-19 cells 4. Activated the AMPK/Nrf2/HO-1 pathway 5. Increased HDL levels and decreased LDL levels 6. Improved the thickness of the retinal layer 7. Decreased retinal cell apoptosis	AMPK/Nrf2/HO-1	[113,114]
Multiple Bioactive Agents	Ginsenoside Rg1, Ginsenoside Rb1	*Panax notoginseng sponins*	MIO-M1 cells/STZ rats	1. Increased retinal inner nuclear layer thickness, reduced acellular capillaries, and attenuated BRB disruption by upregulated claudin-1 and occluding 2. Abrogated microglial cell activation, and reversed leukocyte adhesion by downregulated intercellular molecule-1 and vascular cell adhesion molecule-1 3. Reduced pro-inflammatory factors (TNF-α, IL-6, and IL-1β), and inhibited expressions of p-IKK, p-IκB, p-p65, and nuclear translocation of p65	NF-κB	[14]
	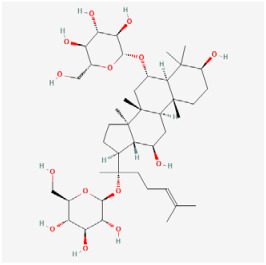 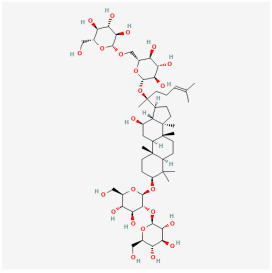					
	60% Edible ethanolic extract of U. davidiana, catechin 7-O-β-D-apiofuranoside 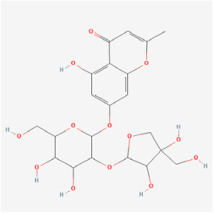	*Ulmus dacidiana*	Human placental pericytes/HMVECs	1. Prevented pericyte apoptosis by blocking the activities of p38 and JNK 2. Increased ZO-1 expression and reduced endothelial permeability by preventing pericyte apoptosis	-	[115]

**Table 2:** Protective effects of natural products in the treatment of DR.

Target	Effects	Compound Name	Natural Product	Category	Reference
Inflammation	Reduced inflammatory factors (IL-1β, IL-6, TNF-α, Interferon-γ, and MCP-1)	Resveratrol	Grape, peanuts, berries	Phenols	[86,87,88,89]
	1. Downregulated inflammatory factors (TNF-α, IL-1β, IL-6, MIC-1, and IL-10) 2. Downregulated NF- kB level	Astaxanthin	*Haematococcus pluvialis*, shrimps	Terpenoids	[40,90]
	Suppressed inflammatory cytokines (TNF-α and IL-1β)	Quercetin	Licorice	Flavonoids	[91,92]
	Suppressed inflammatory cytokines (TNF-α, IL-1β, and IL-6)	Asiaticoside	*Centella asiatica*	Saponins	[95]
	1. Suppressed inflammatory factors (TNF-α, IL-1β, and IL-6) 2. Suppressed TLR4 and NF-kB expressions	Nimbolide	Neem plants	Terpenoids	[98]
	Suppressed inflammatory cytokines (TNF-α, IL-1β, and IL-6)	Argunolic acid	*Cyclocarya paliurus*	Terpenoids	[37]
	1. Reduced pro-inflammatory cytokines (IL-18 and IL-1β) 2. Inhibited NLRP3 inflammasome formation	Palbinone	*Paeonia suffruticosa*	Terpenoids	[99]
	Suppressed inflammatory cytokines (TNF-α, IL-1β, and IL-6)	Tanshinone IIA	*Salvia miltiorrhiza* bunge	Terpenoids	[101]
	Downregulated the expression of inflammasome components (NLRP3 and ASC) along with their downstream targets (caspase-1 and IL-1β)	Tilianin	*Traversia baccharoides Hook.f.*	Flavonoids	[104]
	Suppressed inflammatory cytokines (TNF-α and IL-1β)	Morin	Moraceae family plants	Flavonoids	[105]
	1. Suppressed inflammatory cytokines (TNF-α, IL-1β, and IL-6) 2. Downregulated the expressions of COX-2 and p65	Diosgenin	Fenugreek seeds, wild yam roots	Steroids	[113,114]
	1. Suppressed pro-inflammatory factors (TNF-α, IL-6, and IL-1β) 2. Inhibited the activation of the NF-kB signaling pathway	Ginsenoside Rg1, Ginsenoside Rb1	*Panax notoginseng sponins*	-	[14]
Neovascularization/VEGF	1. Suppressed VEGF and HIF-1α expressions 2. Suppressed neovascularization by inactivation of cell proliferation and migration	Berberine	*Rhizoma Coptidis*	Alkaloids	[31,93,94]
	Reduced VEGF expression	Astragalin	*Astragalus mambranaceus*	Saponins	[96]
	Inhibited tube formation induced by VEGFR2 and BMP4	Glechomanamides B	Salvia	Terpenoids	[49]
	Reduced VEGF expression	Tanshinone IIA	*Salvia miltiorrhiza* bunge	Terpenoids	[101]
	1. Inhibited cell proliferation, migration, and tube formation 2. Suppressed the expressions of VEGF and PGF	Kaempferol	*Kaempferia galanga* L.	Flavonoids	[102,103]
	1. Inhibited cell migration 2. Inhibited HIF-1α and VEGF expressions	Chrysin	*Oroxylum indicum (Linn.) Bentham ex Kurz*	Flavonoids	[50]
	1. Suppressed cell proliferation, migration, and tube formation 2. Inhibited HIF-1α and VEGF expressions 3. Decreased angiogenesis-related expressions of FGF2 and PAI-1	Gambogic acid	Gamboges resin	Flavonoids	[51]
	1. Inhibited cell proliferation 2. Inhibited HIF-1α and VEGF expressions	Fucoidan	Brown algae	Saccharides	[110,111]
	1. Inhibited the secretion of VEGFA 2. Decreased the mRNA expressions of VEGFA and PHD-2 3. Inhibited hypoxia-induced retinal neovascularization	Aloe-emodin	Radix et Rhizoma Rhei	Quinones	[112]
Oxidative stress	Reduced lipid peroxidation level and ROS content	Astragaloside-IV	*Astragalus mambranaceus*	Saponins	[55,84,85]
	1. Enhanced SOD activity and reduced the 8-isoprostane level and GSSG/GSH ratio in both blood and retina tissue 2. Decreased oxidative stress marker 4-HNE	Resveratrol	Grape, peanuts, berries	Phenols	[86,87,88,89]
	1. Regulated GSH, GPx, MDA, and ROS levels 2. Fostered the expressions of HO-1, NQO1, and IL-10 and restrained the expression of NF-kB	Astaxanthin	*Haematococcus pluvialis*, shrimps	Terpenoids	[40,90]
	Increased GSH and antioxidant enzymes’ activities (SOD and CAT)	Quercetin	Licorice	Flavonoids	[91,92]
	Reduced oxidative stress	Berberine	*Rhizoma Coptidis*	Alkaloids	[31,93,94]
	1. Decreased ROS production 2. Increased HO-1 activity	Carnosol	Rosemary	Phenols	[97]
	Decreased MDA and lipid peroxidation levels	Magnolol	*Magoolia officinalis*	Phenols	[56]
	Improved antioxidants’ status by regulating SOD, GSH, SOD/CAT ratio, and GSH/GSSG ratio	Nimbolide	Neem plants	Terpenoids	[98]
	1. Upregulated the HO-1 protein level 2. Downregulated ROS and MDA levels	Argunolic acid	*Cyclocarya paliurus*	Terpenoids	[37]
	1. Enhanced antioxidant activities (SOD, CAT, and GPx) 2. Enhanced the HO-1 expression	Palbinone	*Paeonia suffruticosa*	Terpenoids	[99]
	1. Restored the GSH level 2. Inhibited IDO and consequential changes in Kynurenine metabolites	Andrographolide	*Andrographis paniculate (Burm. F.) Nees*	Terpenoids	[100]
	Decreased ROS levels	Kaempferol	*Hippophae rhamnoides* L.	Flavonoids	[102,103]
	1. Increased Nrf2 and HO-1 expressions and decreased MDA levels 2. Increased SOD, CAT, and GPX levels and decreased MDA levels	Tilianin	*Traversia baccharoides Hook.f*.	Flavonoids	[104]
	1. Improved endogenous antioxidant enzymes’ activity (GPx, CAT, and SOD) 2. Decreased lipid peroxidase levels	Morin	Moraceae family plants	Flavonoids	[105]
	1. Restored GSH activity 2. Decreased thiobarbituric acid-reactive substances and protein carbonyl levels 3. Inhibited AR activity 4. Increased antioxidant enzymes’ activities (SOD, GPx, and CAT)	Isoflavones	*Caesalpinia pulcherrima*	Flavonoids	[108]
	Inhibited ROS generation through Ca^2+^-dependent ERK1/2 signaling pathway	Fucoidan	Brown algae	Saccharides	[110,111]
	Increased SOD and GPX levels and decreased MDA levels	Diosgenin	Fenugreek seeds, wild yam roots	Steroids	[112]
Apoptosis	Decreased cell apoptosis	Astragaloside-IV	*Astragalus mambranaceus*	Saponins	[55,84,85]
	1. Mediated alternations in gene and protein expressions related to apoptosis (p53, Bax, Bcl-2, caspase-3, caspase-9, p38aMAPK, c-Jun N-terminal kinase-1, and extracellular signal-regulated kinase-1) 2. Blocked the increase in CaMKII and phosphor-CaMKII protein levels	Resveratrol	Grape, peanuts, berries	Phenol	[86,87,88,89]
	1. Inhibited retinal pericytes apoptosis 2. Regulated apoptosis-related proteins (Bcl-2, BAX, and caspase-3)	Astaxanthin	*Haematococcus pluvialis*, shrimps	Terpenoids	[40,90]
	1. Attenuated apoptosis by decreasing caspase-3 and cytochrome c levels, and increasing Bcl-2 expression 2. Decreased the expression of BDNF, NGF, TrkB receptor, synaptophysin, and p-Akt 3. Suppressed NF-kB activity	Quercetin	Licorice	Flavonoids	[91,92]
	regulated apoptosis-related proteins (Bcl-2, Bax, caspase-3, and caspase-9)	Asiaticoside	*Centella asiatica*	Saponins	[95]
	Attenuated cell apoptosis and caspase-3 activity	Kaempferol	*Kaempferia galanga* L.	Flavonoids	[102,103]
	Ameliorated apoptosis by inhibiting caspase-9 activity	7,8-Dihydroxyflavone	Citrus, grains, tea	Flavonoids	[106]
	Inhibited retinal cell apoptosis	Epicatechin	Green tea, coconut	Flavonoids	[109]
	1. Suppressed cell apoptosis 2. Regulated apoptosis-related proteins (Bcl-2, BAX, and caspase-3)	Diosgenin	Fenugreek seeds, wild yam roots	Steroids	[113,114]
	1. Prevented pericyte apoptosis by blocking the activities of p38 and JNK 2. Prevented apoptosis-associated protein (capsase-3)	U60E, C7A	*Ulmus dacidiana*	-	[115]
Ferroptosis	Decreased the rate of ferroptosis by inhibiting the expression of miR-138-5p	Astragaloside-IV	*Astragalus mambranaceus*	Saponins	[55,84,85]
Cell phosphorylation	Regulated ERK1/2 phosphorylation	Astragaloside-IV	*Astragalus mambranaceus*	Saponins	[55,84,85]
	Regulated ERK1/2 phosphorylation	Kaempferol	*Kaempferia galanga* L.	Flavonoids	[102,103]
	Regulated AKT and ERK phosphorylation	Chrysin	*Oroxylum indicum (Linn.) Bentham ex Kurz*	Flavonoids	[50]
AGEs accumulation	Decreased AGEs production	Resveratrol	Grape, peanuts, berries	Phenols	[86,87,88,89]
	Decreased AGEs production	Astaxanthin	*Haematococcus pluvialis*, shrimps	Terpenoids	[40,90]
	Reduced AGEs burden	Epicatechin	Green tea	Flavonoids	[109]
MMP	Decreased MMP-2 expression	Chrysin	*Oroxylum indicum (Linn.) Bentham ex Kurz*	Flavonoids	[50]
	Attenuated MMP-9 enzymatic activity	Nobiletin	Citrus plants	Flavonoids	[107]
Autophagy	Activated the AMPK/mTOR/HO-1-regulated autophagy pathway	Arjunolic acid	*Cyclocarya paliurus*	Terpenoids	[37]
Leukocyte adhesion	Inhibited leukocyte adhesion and decreased ICAM-1 expression	Berberine	*Rhizoma Coptidis*	Alkaloids	[31,93,94]
	Downregulated the protein expression of ICAM-1 and VCAM-1	Ginsenoside Rg1, Ginsenoside Rb1	*Panax notoginseng sponins*	-	[14]
